# Combined Immunotherapy in Metastatic Melanoma with Unknown Primary

**DOI:** 10.7759/cureus.5324

**Published:** 2019-08-05

**Authors:** Yasir Khan, Nishanth Thalambedu

**Affiliations:** 1 Internal Medicine, Abington Hospital - Jefferson Health, Abington, USA

**Keywords:** metastatic melanoma with unknown primary, nivolumab, immunotherapy, ipilimumab

## Abstract

Melanoma is skin cancer arising from melanocytes. It may metastasize to unusual areas of the body. Metastatic melanoma with unknown primary (MUP) is relatively uncommon. We present a case of MUP in a 55-year-old male, who underwent adjuvant treatment with combined immunotherapy and showed a good response. We report this case to alert fellow physicians that immunotherapy can be used in MUP with excellent outcomes.

## Introduction

Metastatic melanoma with unknown primary (MUP) is a rare entity. Very little is known about it in the literature. Studies conducted for metastatic melanoma never reported specific subgroup outcomes for MUP [1]. Currently, advanced melanoma is being treated the same irrespective of its subtype. But survival rates have been different, which makes MUP different from metastatic melanoma with known primary (MKP). It was hypothesized that the improved survival in MUP is because of the inherent robust protective immune response of the host [2-3]. That being said, it is not quite sure how immunotherapy works for MUP. Clinicians do not have any specific guidelines to use immunotherapy in MUP because there were no studies done on this subset of patients. We present a case of metastatic CNS melanoma with unknown primary, which was treated with adjuvant nivolumab and ipilimumab and showed a significant response. Nonetheless, more research needs to be done on this subset to have a clear understanding.

## Case presentation

A 57-year-old-male, nonsmoker, presented with left upper extremity weakness, slurred speech, and facial droop that began 12 hours before presentation. His past medical history was significant for prostate cancer that was treated with radical prostatectomy six years ago without any hormonal or radiotherapy. On physical exam, his vitals were hemodynamically stable. On neuro exam, there was a left-sided facial droop along with left upper extremity weakness of ⅘ strength with intact sensations. His cardiac and pulmonary exam was within normal limits.

Investigations

**Figure 1:**
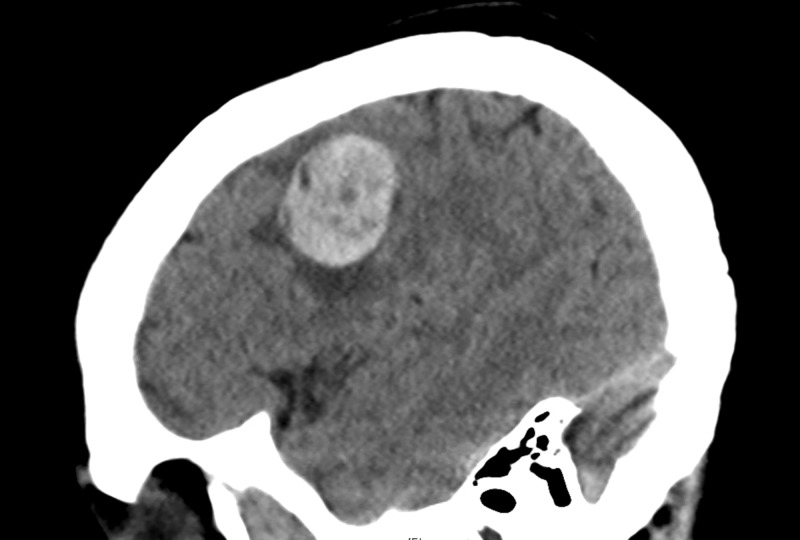
CT scan brain

**Figure 2:**
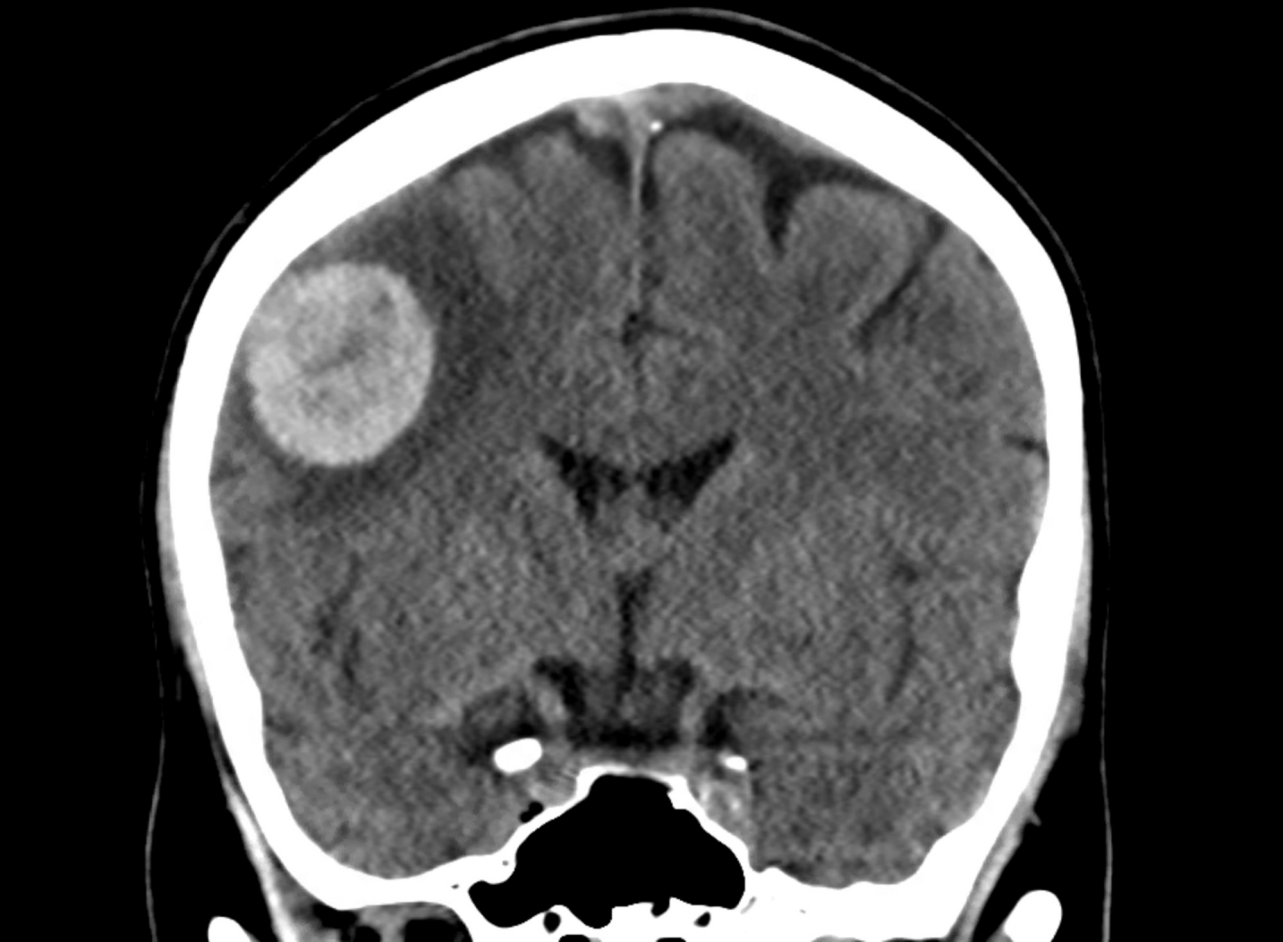
CT scan brain

Pertinent blood tests showed prostate-specific antigen (PSA) levels <0.02. Radiological investigations, including computed tomography (CT) scan brain, showed an acute 2.5 cm right frontal intraparenchymal hematoma with mild white matter edema (Figures 1-2).

**Figure 3:**
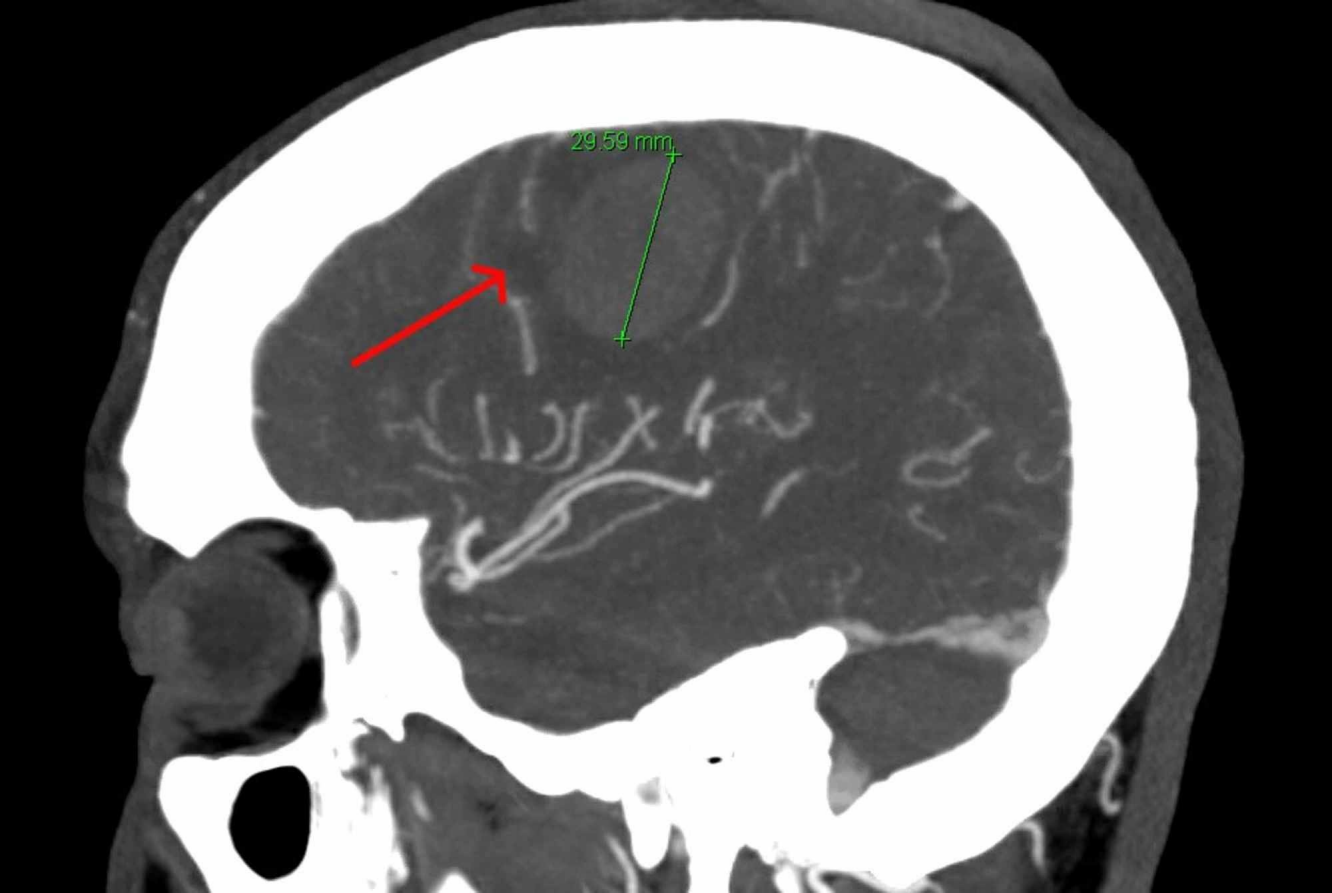
CT angiogram head and neck

CT angiogram of the head and neck revealed a 2.7 x 2.5 x 3 cm lateral convexity intracerebral hematoma in the posterior right frontal lobe with associated vasogenic edema without significant mass effect (Figure 3).

**Figure 4:**
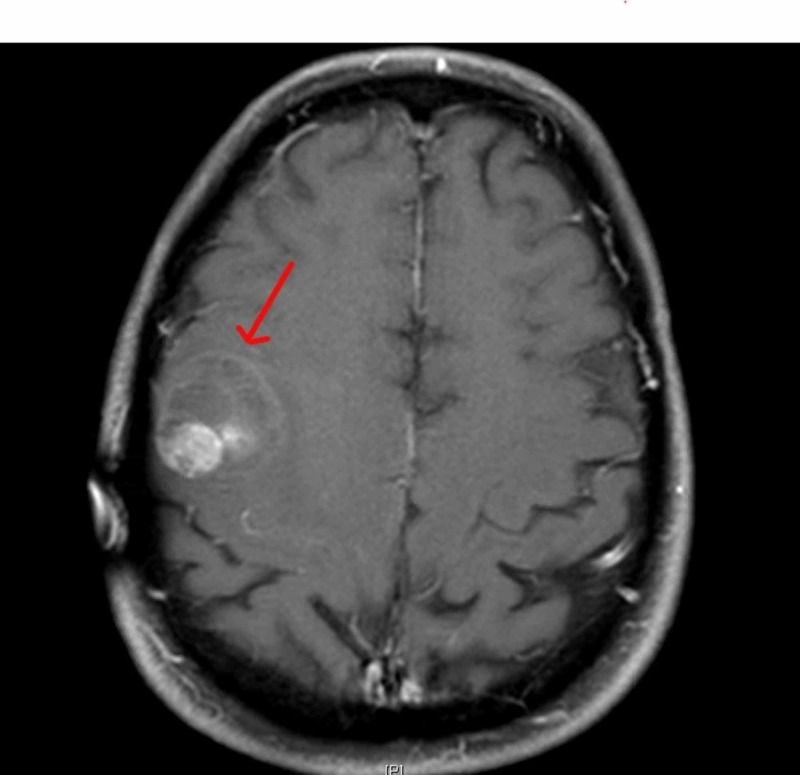
MRI brain

There is no detectable aneurysm or vascular malformation with no evidence of atherosclerotic disease or dissection and no evidence of an embolus. We did a magnetic resonance imaging (MRI) scan of the brain with and without contrast, which revealed a solitary right frontal lobe solid/cystic hemorrhagic mass with fluid-fluid level and surrounding vasogenic edema (Figure 4).

**Figure 5:**
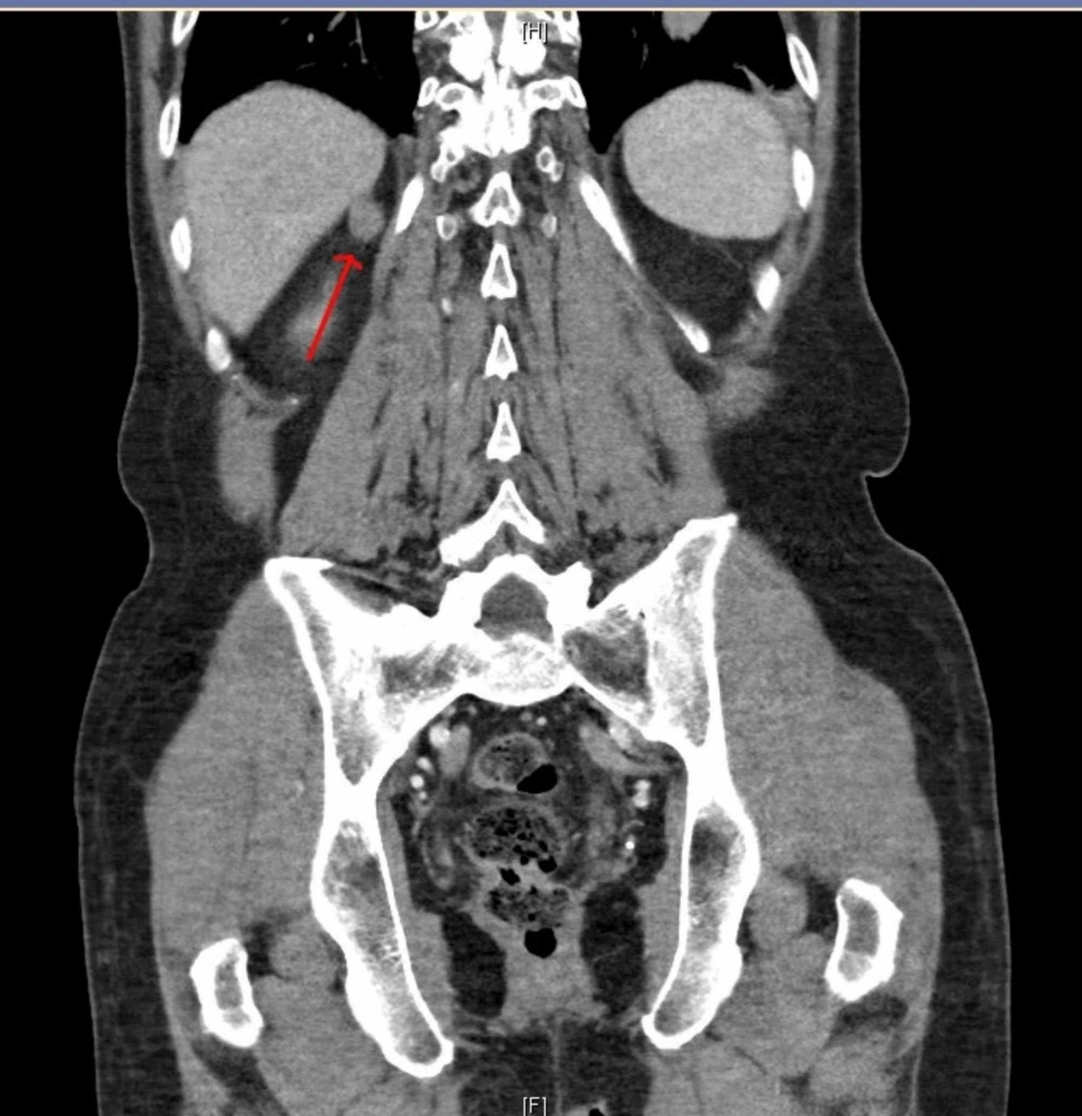
CT chest abdomen and pelvis

**Figure 6:**
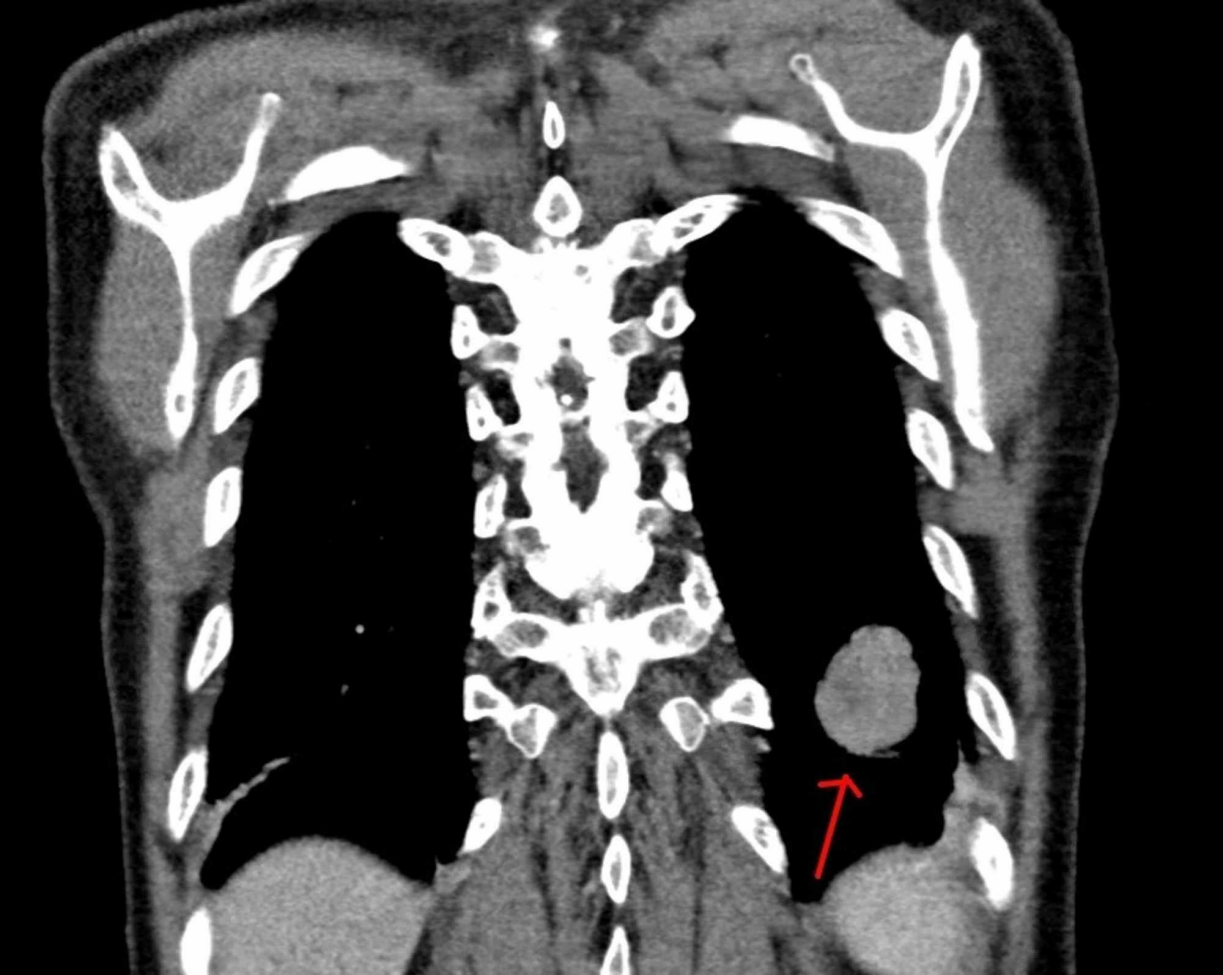
CT chest abdomen and pelvis

We also performed CT chest abdomen and pelvis, and it showed a 4 (CC) x 2.8 (AP) by 2.8 (TV) cm left lower lobe pulmonary mass. It also showed a soft tissue nodule posterior to the liver, but it was unclear if it was arising from the liver (Figures 5-6).

Differential diagnosis

At this time, the main differentials were prostate cancer with central nervous system (CNS) and lung metastases or primary bronchogenic carcinoma with CNS met or a primary CNS tumor.

Treatment

He was initially started on dexamethasone for vasogenic edema and Keppra for seizure prophylaxis. The patient underwent image-guided stereotactic right frontal craniotomy for excision of the tumor. The tumor was sent for pathology, which was positive for S100, SOX10, and MelanA and negative for panCK, p40, and GFAP. This is consistent with metastatic melanoma. The tumor sample was sent for molecular testing, which was negative for BRAF gene mutations.

Further investigations

The patient underwent a positron emission tomography (PET) scan, which showed two lesions outside of the CNS. One was a 2 cm mass in the left lower lobe of the lung with a standardized uptake value (SUV) of 9.3 and the second one was a 1.2 cm right retroperitoneal implant with an SUV of 5.6. Both these lesions were consistent with possible sites of metastatic melanoma, but this was not confirmed with a biopsy. He underwent a thorough dermatological exam that did not yield any lesion.

Outcome and follow-up

The patient underwent Cyberknife (Accuray Incorporated, California, United States) stereotactic radiosurgery (SRS) to the right frontal pole (FP) surgical cavity. He was started on nivolumab and ipilimumab. He finished four cycles. Repeat MRI and CT scan showed no evidence of recurrence or new metastasis.

## Discussion

Melanoma is an aggressive malignancy arising from melanocytes [4]. It is the fifth most common cancer in men and women in the US and metastasizes to unusual areas of the body. Melanoma is a deadly disease that is reflected by an estimation of 96,000 new cases and around 8000 deaths from metastatic melanoma in the US in 2019 [5]. Metastatic melanoma is broadly classified into two types, melanoma with known primary (MKP) and melanoma with unknown primary (MUP). MKP constitutes up to 97% of all metastatic melanomas, with the skin being the most common primary site, and MUP constitutes the balance 3% with lymph nodes being the most common site followed by subcutaneous tissues and visceral organs [6]. Das Gupta first described MUP in the literature in 1963 [7].

Very little is known about MUP and its biology. There are multiple hypotheses postulated to explain MUP. However, the two main hypotheses are as follows: (i) complete spontaneous regression of primary melanoma after metastasis occurred [8], and (ii) primary origin from ectopic melanocytes in lymph nodes or viscera [9]. MUP has been rarely reported in clinical trials. Multiple trials for immunotherapy treatments for advanced melanoma never reported response rates specific to the MUP subgroup population. One of the explanations could be because of its low incidence [1].

According to American Joint Committee on Cancer (AJCC), MUP in subcutaneous tissues, soft tissues, and lymph nodes are diagnosed with stage III disease, and MUP in visceral organs is diagnosed with stage IV disease [10]. Our patient had melanoma in the CNS along with two other lesions in the lung and liver, thus confirming the diagnosis of stage IV disease.

Studies have shown improved survival for stage IV MUP with aggressive management. In a study by Lee et al., the median overall survival (OS) and five-year OS rates were higher for MUP than stage-matched MKP, and this was statistically significant. They also concluded that stage IV MUP with an innate survival advantage are excellent candidates for aggressive management [2]. Also, in a meta-analysis by Bae et al., patients with MUP were found to have better overall survival than MKP in both stage III and stage IV. They also concluded that the autoimmune response of MUP patients might help them to have better survival [3]. Our patient underwent resection, followed by Cyberknife SRS for the right FP surgical cavity.

Adjuvant therapy for a metastatic disease includes immunotherapy and targeted therapy. Immunotherapy options include ipilimumab, which is an anti-CTLA4 antibody; nivolumab, an anti-PD-1 monoclonal antibody; pembrolizumab; and another anti-PD-1 antibody. Combination therapies involving the anti-CTLA-4 antibody and anti-PD-1 antibody have shown prolonged progression-free survival (PFS) and overall OS in patients with advanced melanoma [11-14]. A recent study by Twabi et al. combined nivolumab and ipilimumab and had clinically meaningful intracranial efficacy, concordant with extracranial activity, in patients with melanoma who had untreated brain metastases [15]. Our patient biopsy specimen was negative for any mutation for targeted therapy, so he was started on combined nivolumab and ipilimumab and showed a good clinical response.

## Conclusions

In conclusion, MUP is a relatively rare, understudied, and under-reported entity. Further research is required to understand its biology. Currently, multiple studies have shown better survival for MUP than for stage-matched MKP. Immunotherapy shows a good clinical response in MUP patients. Advanced melanoma should be aggressively treated whenever possible.

## References

[1] Utter K, Goldman C, Weiss SA (2017). Treatment outcomes for metastatic melanoma of unknown primary in the new era: a single-institution study and review of the literature. Oncology.

[2] Lee CC, Fairies MB, Wanek LA, Morton DL (2009). Improved survival for stage IV melanoma from an unknown primary site. J Clin Oncol.

[3] Bae JM, Choi YY, Kim DS (2015). Metastatic melanomas of unknown primary show better prognosis than those of known primary: a systematic review and meta-analysis of observational studies. J Am Acad Dermatol.

[4] Chin L, Garraway LA, Fisher DE (2006). Malignant melanoma: genetics and therapeutics in the genomic era. Genes Dev.

[5] Jemal A, Siegel R, Ward E, Hao Y, Xu J, Murray T, Thun MJ (2008). Cancer statistics. CA Cancer J Clin.

[6] Kamposioras K, Pentheroudakis G, Pectasides D, Pavlidisc N (2011). Malignant melanoma of unknown primary site. To make the long story short. A systematic review of the literature. Crit Rev Oncol Hematol.

[7] Dasgupta T, Bowden L, Berg JW (1963). Malignant melanoma of unknown primary origin. Surg Gynecol Obstet.

[8] Smith JL Jr, Stehlin JS Jr (1965). Spontaneous regression of primary malignant melanomas with regional metastases. Cancer.

[9] Bankar S, Patkar S, Desai S, Shrikhande SV (2015). Unusual presentation of melanoma of unknown primary origin: a case report and review of literature. J Cancer Res Ther.

[10] Gershenwald JE, Scolyer RA, Hess KR (2017). Melanoma staging: evidence‐based changes in the American Joint Committee on Cancer Eighth Edition Cancer Staging Manual. CA Cancer J Clin.

[11] Robert C, Schachter J, Long GV (2015). Pembrolizumab versus ipilimumab in advanced melanoma. N Engl J Med.

[12] Larkin J, Chiarion-Sileni V, Gonzalez R (2015). Combined nivolumab and ipilimumab or monotherapy in previously untreated melanoma. N Engl J Med.

[13] Postow MA, Chesney J, Pavlick AC (2015). Nivolumab and ipilimumab versus ipilimumab in untreated melanoma. N Engl J Med.

[14] Wolchok JD, Chiarion-Sileni V, Gonzalez R (2017). Overall survival with combined nivolumab and ipilimumab in advanced melanoma. N Engl J Med.

[15] Tawbi HA, Forsyth PA, Algazi A (2018 Aug). Combined nivolumab and ipilimumab in melanoma metastatic to the brain. N Engl J Med.

